# Joint Molecule Resolution Requires the Redundant Activities of MUS-81 and XPF-1 during *Caenorhabditis elegans* Meiosis

**DOI:** 10.1371/journal.pgen.1003582

**Published:** 2013-07-18

**Authors:** Nigel J. O'Neil, Julie S. Martin, Jillian L. Youds, Jordan D. Ward, Mark I. R. Petalcorin, Anne M. Rose, Simon J. Boulton

**Affiliations:** 1 Department of Medical Genetics, Faculty of Medicine, University of British Columbia, Vancouver, British Columbia, Canada; 2 DNA Damage Response Laboratory, London Research Institute, Cancer Research UK, South Mimms, United Kingdom; National Cancer Institute, United States of America

## Abstract

The generation and resolution of joint molecule recombination intermediates is required to ensure bipolar chromosome segregation during meiosis. During wild type meiosis in *Caenorhabditis elegans*, SPO-11-generated double stranded breaks are resolved to generate a single crossover per bivalent and the remaining recombination intermediates are resolved as noncrossovers. We discovered that early recombination intermediates are limited by the *C. elegans* BLM ortholog, HIM-6, and in the absence of HIM-6 by the structure specific endonuclease MUS-81. In the absence of both MUS-81 and HIM-6, recombination intermediates persist, leading to chromosome breakage at diakinesis and inviable embryos. MUS-81 has an additional role in resolving late recombination intermediates in *C. elegans*. *mus-81* mutants exhibited reduced crossover recombination frequencies suggesting that MUS-81 is required to generate a subset of meiotic crossovers. Similarly, the Mus81-related endonuclease XPF-1 is also required for a subset of meiotic crossovers. Although *C. elegans gen-1* mutants have no detectable meiotic defect either alone or in combination with *him-6*, *mus-81* or *xpf-1* mutations, *mus-81;xpf-1* double mutants are synthetic lethal. While *mus-81;xpf-1* double mutants are proficient for the processing of early recombination intermediates, they exhibit defects in the post-pachytene chromosome reorganization and the asymmetric disassembly of the synaptonemal complex, presumably triggered by crossovers or crossover precursors. Consistent with a defect in resolving late recombination intermediates, *mus-81; xpf-1* diakinetic bivalents are aberrant with fine DNA bridges visible between two distinct DAPI staining bodies. We were able to suppress the aberrant bivalent phenotype by microinjection of activated human GEN1 protein, which can cleave Holliday junctions, suggesting that the DNA bridges in *mus-81; xpf-1* diakinetic oocytes are unresolved Holliday junctions. We propose that the MUS-81 and XPF-1 endonucleases act redundantly to process late recombination intermediates to form crossovers during *C. elegans* meiosis.

## Author Summary

Meiotic recombination generates joint molecules that ensure chromosomes segregate correctly. Failure to generate or resolve joint molecules can have profound effects on fertility and on the viability of resulting progeny. The generation and resolution of joint molecules is carefully regulated. Generation of joint molecules is highly similar across a broad range of organisms, from yeast to mammals. Yet, the resolution of the resultant joint molecules varies across organisms, with helicases and endonucleases contributing to varying extents in different organisms. We used the genetically tractable model organism, *Caenorhabditis elegans (C. elegans)* to uncover redundancies between joint molecule processing proteins. Specifically, we investigated the contribution of the *C. elegans* BLM helicase ortholog, HIM-6, and the endonucleases MUS-81, XPF-1, GEN-1 and EXO-1 to the resolution of meiotic joint molecules. We found that MUS-81 and HIM-6 act redundantly to resolve joint molecules early in meiosis, presumably to form noncrossovers. Late in meiosis, MUS-81 and XPF-1 act redundantly to resolve joint molecules to form crossovers. When both MUS-81 and XPF-1 are absent, joint molecules are not resolved, resulting in disorganized chromosomes in the oocyte and embryonic death. Joint molecules in *mus-81;xpf-1* animals are rescued by microinjection of the human GEN1 protein, indicating these intermediates are Holliday junctions.

## Introduction

Meiotic recombination generates chiasmata that join homologous chromosomes together to ensure proper meiotic chromosome segregation. The efficient generation and resolution of joint molecules (JM) is essential for meiosis; therefore, JM formation and resolution is carefully regulated. In most organisms, meiotic recombination is initiated by the generation of Spo11-induced double strand breaks (DSBs). DSBs are resected to produce a 3′ single-stranded stretch of DNA onto which Rad51 is loaded, forming a nucleoprotein filament. Rad51 catalyzes invasion of the homologous chromosome and JM intermediates physically linking homologous chromosomes are formed (reviewed in [1]). JMs must be resolved before homologs segregate at meiosis I. JMs can be resolved to form crossover (CO) products in which flanking markers are exchanged, or they can be resolved to form non-crossover (NCO) products. The overall progression of JM resolution appears to be similar in diverse organisms, however the proteins and their relative involvement in JM resolution vary from species to species. Thus, the same initiating lesion (Spo11-induced DSB) is repaired through diverse mechanisms. Study of meiotic DSB repair in a range of organisms illuminates the modularity of repair and how different organisms have evolved to favor distinct endonucleases to repair Spo11 generated DSBs.

There are two main pathways that process meiotic JMs to COs, which are utilized to varying extents in different organisms. Much of what we know about meiotic crossover resolution comes from studies in budding yeast and fission yeast. The predominant pathway in budding yeast involves the synaptonemal complex-associated ZMM (*Z*ip1/2/3/4, Msh4/5, Mer3) proteins. It has been proposed that the ZMM proteins protect recombination intermediates (RIs) from NCO resolution, ensuring CO resolution of ZMM-associated RIs [2], [3]. ZMM-dependent COs are subject to crossover interference, which occurs when the presence of one CO decreases the probability that another CO will occur nearby. A second ZMM-independent pathway is characterized by the structure specific endonuclease Mus81/Mms4 that resolves RIs to either CO or NCO outcomes [4], [5]. These ZMM-independent COs are not subject to crossover interference.

Fission yeast, which lack both a synaptonemal complex and ZMM proteins, represent an extreme case in which all COs are ZMM-independent and are resolved by Mus81/Eme1. Loss of Mus81 in *S. pombe* results in spore inviability due to meiotic chromosome segregation defects and a profound decrease in the frequency of COs [6], [7]. Consistent with Mus81 being the major meiotic Holliday junction (HJ) resolvase in fission yeast, the meiotic chromosome segregation defects of Mus81 mutants can be rescued by the expression of the bacterial HJ resolvase RusA [7] or by the expression of the human Holliday junction resolvase GEN1 [8].

Loss of Mus81 in budding yeast, which has a synaptonemal complex and ZMM-proteins, results in only a minor reduction in spore viability and a modest decrease in the frequency of COs [9]. These data are consistent with a model in which Mus81 is only responsible for ZMM-independent COs and that ZMM-dependent COs predominate in budding yeast [5]. This bias towards the ZMM-dependent CO pathway in budding yeast is mediated by the BLM-helicase homolog, Sgs1 [10]–[13]. Sgs1 is thought to prevent the accumulation of JMs by channeling most double strand breaks towards NCO resolution and ensuring that remaining DSBs are associated with ZMM-proteins and resolve as COs [10], [13]. When Sgs1 is absent, JMs accumulate and require Mus81 for resolution. Cells lacking both Sgs1 and Mus81 during meiosis accumulate JMs and cannot segregate at meiosis I [11], [12].

Although, loss of Mus81 does not greatly affect meiotic segregation in budding yeast, it appears that the Yen1 and Slx1/Slx4 endonucleases act redundantly with Mus81 to resolve persistent JMs. Loss of Yen1 alone does not result in meiotic phenotypes whereas *mus81 yen1* double mutants exhibit a profound decrease in spore viability due to a failure of JM resolution and chromosome segregation at meiosis I [14]. Similarly, the Slx4/Slx1 endonuclease processes some RIs in the absence of Mus81 [10], [13].

The structure-specific endonucleases are also critical for crossing over in more complex organisms. In Drosophila, MUS81 does not appear to play a role in the formation of COs [15]. Most COs are catalyzed by the Mus81-related structure specific endonuclease MEI-9 (fly nucleotide excision repair endonuclease XPF ortholog), and MUS312 (fly SLX4 ortholog). Loss of function of either MEI-9 or MUS312 results in a severe decrease in crossing-over [16] suggesting that MUS312 and MEI-9 resolve meiotic HJ intermediates. The MUS81 and XPF endonucleases also play a role in mouse meiosis. Although *Mus81* knockout mice are viable and fertile [17], [18], MUS81 appears to be required for the repair of at least some meiotic DSBs during murine meiosis. *Mus81-/-* male mice exhibit significant meiotic defects in the germ line: mature sperm are depleted, MLH1 foci, which mark ZMM-dependent crossovers, are increased, and a subset of meiotic DSBs is not repaired [19]. Similar to *Mus81* mutant mice, mice lacking ERCC1 (the binding partner of XPF) or BTBD12 (Slx4 ortholog) exhibit sperm defects, persistent unrepaired DSBs in the germ line, and increased MLH1 foci [20], [21].

*C. elegans* is a powerful model for study of metazoan meiotic DSB repair. The germ line is temporally and spatially polarized with respect to meiotic progression, allowing detection of subtle but significant alterations in the kinetics of repair events. Synaptonemal complex formation and DSB induction are independent during *C. elegans* meiosis, allowing separation of homolog pairing and DSB repair. Finally, crossover interference is incredibly robust in *C. elegans* with only a single CO occurring per pair of homologous chromosomes. In *C. elegans*, it has been proposed that all COs are ZMM-dependent [22] and as such would not require MUS-81 or related structure-specific endonucleases for resolution. However, the study *of rtel-1* anti-recombinase mutants revealed that there are two classes of COs in *C. elegans*: ZMM-dependent COs; and ZMM-independent COs, which require MUS-81 for resolution [23]. Other structure-specific endonucleases also play a role in CO formation in *C. elegans*; loss of XPF-1 (XPF/MEI-9 ortholog) results in a decrease in the number of COs compared to wild type animals. Furthermore, loss of both MUS-81 and HIM-18, the *C. elegans* Slx4 ortholog, results in an increase in mitotic and meiotic RIs [24]. Together, these data suggest that MUS-81 and HIM-18 play overlapping, non-redundant roles in processing RIs. Unlike MUS-81, XPF-1, or HIM-18, and in contrast to yeast, the related endonuclease GEN-1 (Yen1 ortholog) does not appear to have a role during *C. elegans* meiosis [25]. What remains unclear is the relative contribution of each of the structure-specific endonucleases to CO formation and where in the CO pathway each endonuclease functions. Are the endonucleases acting early to process RIs to NCO outcomes or are they acting late to resolve RIs as COs?

Here, we investigated the relative roles of structure-specific endonucleases in processing RIs during meiosis in *C. elegans* with the goal of identifying the enzyme(s) responsible for processing JMs to produce COs. Unexpectedly, we found that MUS-81 performs both early and late roles in processing RIs during meiosis I in *C. elegans* and that its loss results in an overall decrease in the total number of COs. We show that MUS-81 and HIM-6 (Sgs1 homolog) act early during pachytene to limit the accumulation and persistence of RAD-51-associated RIs during meiosis, a defect that also manifests as chromosome fragmentation at diakinesis. Surprisingly, we found that MUS-81 and XPF-1 endonucleases, but not GEN-1 or EXO-1, act redundantly to process late stage JMs to form COs. Loss of both MUS-81 and XPF-1 resulted in defective CO maturation and persistent JMs at diakinesis, which could be rescued by germline injection of the human Holliday junction resolvase GEN1. As human GEN1 is able to cleave HJs *in vitro*, these results strongly suggest that the persistent JMs in *mus-81; xpf-1* double mutants are HJs. Together, these data support a redundant role for MUS-81 and XPF-1 in processing HJ intermediates to produce interhomolog crossovers in *C. elegans*.

## Results

### Loss of *mus-81* does not result in obvious meiotic defects

**Figure 1:**
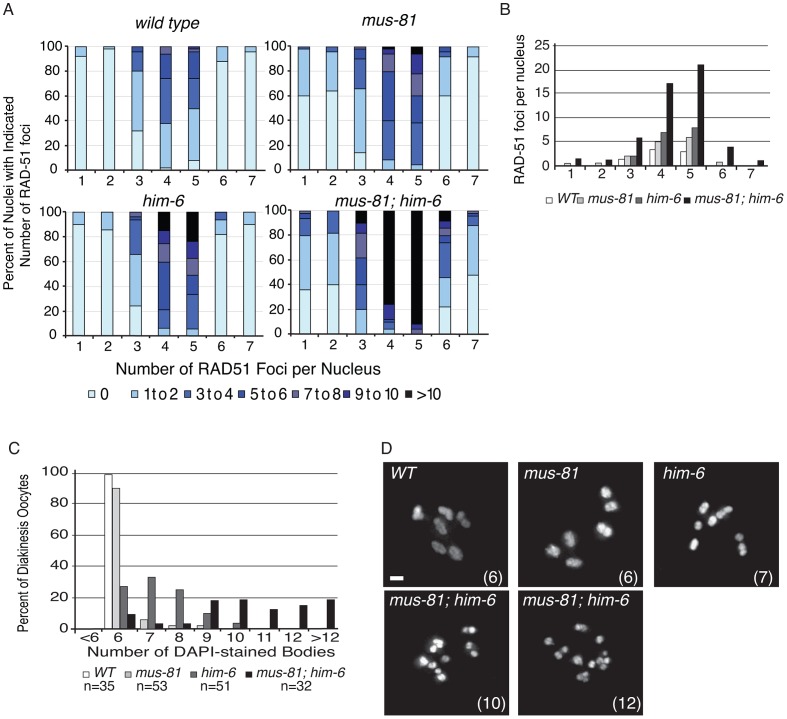
MUS-81 and HIM-6 prevent the accumulation of recombination intermediates and chromosome instability. (A) Distribution of RAD-51 foci in WT, *mus-81*, *him-6*, and *mus-81; him-6* animals. Zone definitions: 1 Early mitotic 2 Late mitotic 3 Transition 4 Early pachytene 5 Mid pachytene 6 Late pachytene 7 Diplotene. (B) Mean number of RAD-51 foci per nucleus from A. (C) Average number of DAPI-stained bodies in diakinesis oocytes of WT, *mus-81*, *him-6*, and *mus-81; him-6* animals. (D) Representative images of DAPI-stained bodies in diakinesis oocytes of WT, *mus-81*, *him-6*, and *mus-81; him-6* animals. Bars 2 µm.

To examine the function of MUS-81 during meiosis in *C. elegans*, we characterized the meiotic phenotype of a *mus-81* null mutant [26]. *C. elegans mus-81(tm1937)* mutants exhibited no obvious phenotypes attributed to meiotic defects such as a high frequency of embryonic inviability or an increased frequency of XO males. *mus-81* mutants had reduced brood sizes (142+/−19, approximately 50% of wild type, Student's *t*-test p<0.005) (Table 1). *mus-81* brood sizes were variable, ranging from rare animals that were completely sterile (1/20 broods scored) to animals that produced more than 200 progeny (5/20 broods scored). The brood size in *C. elegans* is dictated by the number of viable sperm, so defects in the germ line that lead to reduction in the number of viable sperm could result in a smaller brood size. A checkpoint in the *C. elegans* female germ line senses DNA damage or persistent recombination intermediates, triggering apoptosis of the damaged nuclei [27], [28]. To determine if this apoptotic checkpoint protects the *mus-81* germ line by removing defective nuclei before they develop into oocytes, we constructed *mus-81(tm1937); ced-4(n1162)* double mutants. *ced-4* is essential for the initiation of apoptosis in *C. elegans* [29]. *mus-81; ced-4* double mutants had a significantly reduced brood when compared to either *ced-4* or *mus-81* single mutants (Table 1, Student's *t*-Test p<0.01) and 4/10 lines were completely sterile suggesting that the apoptotic checkpoint removes nuclei with DNA damage or aberrant meiotic recombination intermediates in the *mus-81* mutant. This observation together with the reduced brood suggested that there are defects in the *mus-81* germ line. We next assayed the distribution of early RIs in the *C. elegans* germ line using an antibody that recognizes the recombination protein RAD-51. In wild type animals, SPO-11-dependent RAD-51 foci are visible in early pachytene and are resolved by late pachytene (Figure 1; Figure S1; Figure S2). In *mus-81* animals the average number of RAD-51 foci was slightly but significantly increased in all zones except diplotene when compared to wild type animals (Student's *t*-test p<0.05). Apart from a small but significant increase of RAD-51 foci in the mitotic zone (average 0.54 *mus-81*, 0.05 WT) and the persistence of foci in late pachytene, the same general pattern of RAD-51 staining was observed in *mus-81(tm1937)* and wild type backgrounds (Figure 1A, B). Consistent with this observation, *mus-81* mutants did not exhibit high levels of chromosome non-disjunction or fragmentation in diakinetic oocytes (Figure 1C, D).

**Table 1:** Plate phenotypes.

Genotype	Brood Size	% inviable embryos	% Males
N2 wild type	271±13	0.1±0.1	0.2±0.1
*mus-81(tm1937)*	142±19	7.6±10.6	0.6±0.1
*him-6(ok412)*	200±15	53.1±6.0	10.9±0.8
*gen-1(tm2940)*	213±13	0.3±0.1	0.1±0.1
*exo-1(tm1842)*	218±16	0.9±0.2	0.1±0.1
*xpf-1(e1487)*	242±11	8.9±2.6	2.2±0.5
*ced-4(n1162)*	176±30	1.2±0.4	0
*mus-81; ced-4*	51±23	27.5±7.6	0
*mus-81; him-6*	22±5	99.7±0.2	0
*mus-81; gen-1*	125±15	17.9±10.4	0.3±0.2
*mus-81; exo-1*	129±16	38.8±8.5	0.4±0.1
*mus-81; xpf-1*	93±13	74.2±11.6	DNC
*him-6; gen-1*	101±11	56.1±2.3	13.5±1.0
*him-6; exo-1*	120±22	83.5±7.2	15.6±0.6
*him-6; xpf-1*	126±26	71±6.5	14.5±3.9
*xpf-1; gen-1*	129±23	14.4±1.9	3.5±0.9

phenotypes ± SEM.

DNC, Did not count.

### MUS-81 and HIM-6, the *C. elegans* BLM ortholog, limit recombination intermediates

In yeast, loss of Mus81 and the helicase Sgs1 confers synthetic lethality as a result of the accumulation of unresolved RIs [7], [9], [30], [31]. Meiosis-specific mutant alleles of *mus81* and *sgs1* indicate that Sgs1 limits JMs and that, in the absence of Sgs1, Mus81 resolves these structures to prevent the accumulation or persistence of JMs during meiosis [11], [12]. To test whether a similar relationship exists in *C. elegans*, we characterized the formation of RIs in animals lacking MUS-81 and the *C. elegans* Sgs1 helicase ortholog HIM-6 [32]. Similar to yeast *mus81 sgs1* double mutants, *C. elegans mus-81; him-6* double mutants exhibited a severely reduced brood size and ∼100% embryonic lethality (Table 1). DAPI staining revealed that germline nuclei progression in *mus-81; him-6* double mutants was grossly normal with nuclei progressing from the mitotic zone to pachytene and finally diakinesis (Figure S1, Figure S2). To further examine this phenotype we stained germ lines with an anti-RAD-51 antibody to monitor the distribution of early meiotic RIs. Both *mus-81* and *him-6* single mutants exhibited increased RAD-51-associated RIs in mid-pachytene compared to wild type animals (5.94 and 7.92 respectively; WT 2.96, Student's *t*-Test p<0.01). However, the *mus-81; him-6* double mutant accumulated significantly more RIs in mid-pachytene (21.1 per nucleus, Student's *t*-test p<0.01 compared to either single mutant) than would be predicted for the additive effect of the two mutations (Figure 1A, B). Consistent with the generation of large numbers of persistent RIs, *mus-81; him-6* double mutant animals exhibited evidence of chromosome breakage with significantly more DAPI staining bodies in diakinetic oocytes than would be predicted for the additive effect of the two mutants (Figure 1C, D). Approximately 20% of oocytes contained more than 12 DAPI staining bodies and many of the DAPI-stained bodies were very small, suggesting that at least some of these DAPI staining bodies represented chromosome fragments rather than loss of chiasmata between homologs, which would produce 12 univalents. The increased RAD-51 foci in the *mus-81; him-6* double mutant coincided with the meiotic zones in which SPO-11 is active and Agostinho *et al.* [33] demonstrated that the chromosome fragmentation phenotype of *mus-81; him-6* is SPO-11-dependent, Therefore, these RAD-51 foci likely result from SPO-11 generated DSBs, though it is formally possible that these foci resulted from DNA damage arising in the transition zone and early pachytene. Collectively, these data support roles for HIM-6 and MUS-81 in limiting early RIs during meiosis.

### MUS-81 is required for wild type levels of meiotic crossovers

**Figure 2:**
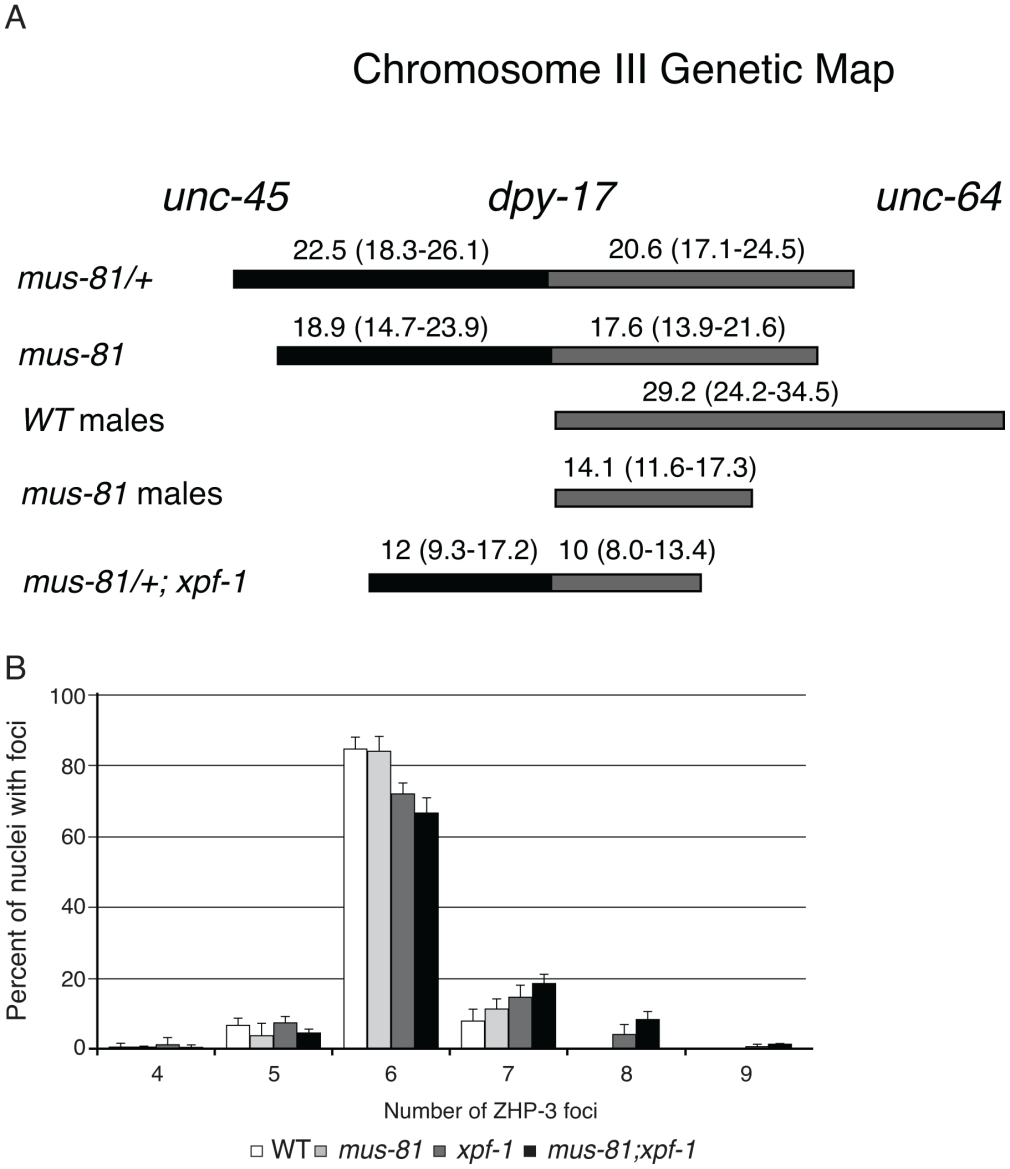
MUS-81 and XPF-1 are required for wild type frequency of crossovers. (A) A schematic depicting the effect of *mus-81* and *xpf-1* mutations on crossover frequency on chromosome III. Confidence intervals (95% CI) shown in brackets were calculated using the statistics of Crow and Gardner [51]. (B) Histograph plotting the average number of ZHP-3 foci in late pachytene nuclei. Error bars are standard error of the mean.

The increased persistent RIs in *mus-81; him-6* double mutants raised the possibility that MUS-81 resolves RIs in the *C. elegans* germ line. Previously, we demonstrated that MUS-81 is required in *rtel-1* mutants to resolve supernumerary meiotic RIs to produce COs [23]. In the absence of MUS-81 in *rtel-1* mutants, large numbers of RAD-51 foci persist into late pachytene [23], which is similar to what we observed in the *mus-81; him-6* double mutant. To determine if MUS-81 played a role in the formation of endogenous COs, we used visible genetic markers to measure recombination frequency in two genetic intervals, *unc-45 dpy-17* and *dpy-17 unc-64*, that span 48.8 map units of chromosome III (approximately 98% of the genetic length). Unexpectedly, recombinant progeny were reduced in both intervals in *mus-81* mutants compared to *mus-81/+* heterozygotes (Figure 2A), demonstrating that MUS-81 promoted meiotic CO generation. The apoptotic checkpoint removes nuclei with DNA damage or meiotic defects and is only active in the female germ line [27], [28], allowing us to test whether this checkpoint was ameliorating the *mus-81* mutant phenotypes. We therefore measured recombination frequencies between *dpy-17* and *unc-64* in *mus-81* males. *mus-81* mutant males exhibited a much greater effect on recombination distances than that observed for hermaphrodites (WT males 29.2 [95% CI 24.2–34.5], *mus-81* males 14.1 [95% CI 11.6–17.3]). The activity of the apoptotic checkpoint in the female germ line could explain the differences in CO frequency observed in our data compared to those reported by Saito *et al.* [34] and Agostinho *et al.* [33], who used a method that only measured COs in oocytes.

The recombination frequency phenotype of *mus-81* mutants was surprising as COs in *C. elegans* require MSH-4/MSH-5 (ZMM-dependent) and are subject to strong crossover interference (Class I crossovers) and therefore would not be expected to require MUS-81 for resolution. Moreover, *mus-81* mutants exhibited a near wild type number of ZHP-3 foci, which mark emerging COs [35] and suggested that MUS-81 acts after ZHP-3 foci formation (Figure 2B, Figure S3).

### *mus-81* is synthetic lethal with loss of the related structure-specific endonuclease *xpf-1*

**Figure 3:**
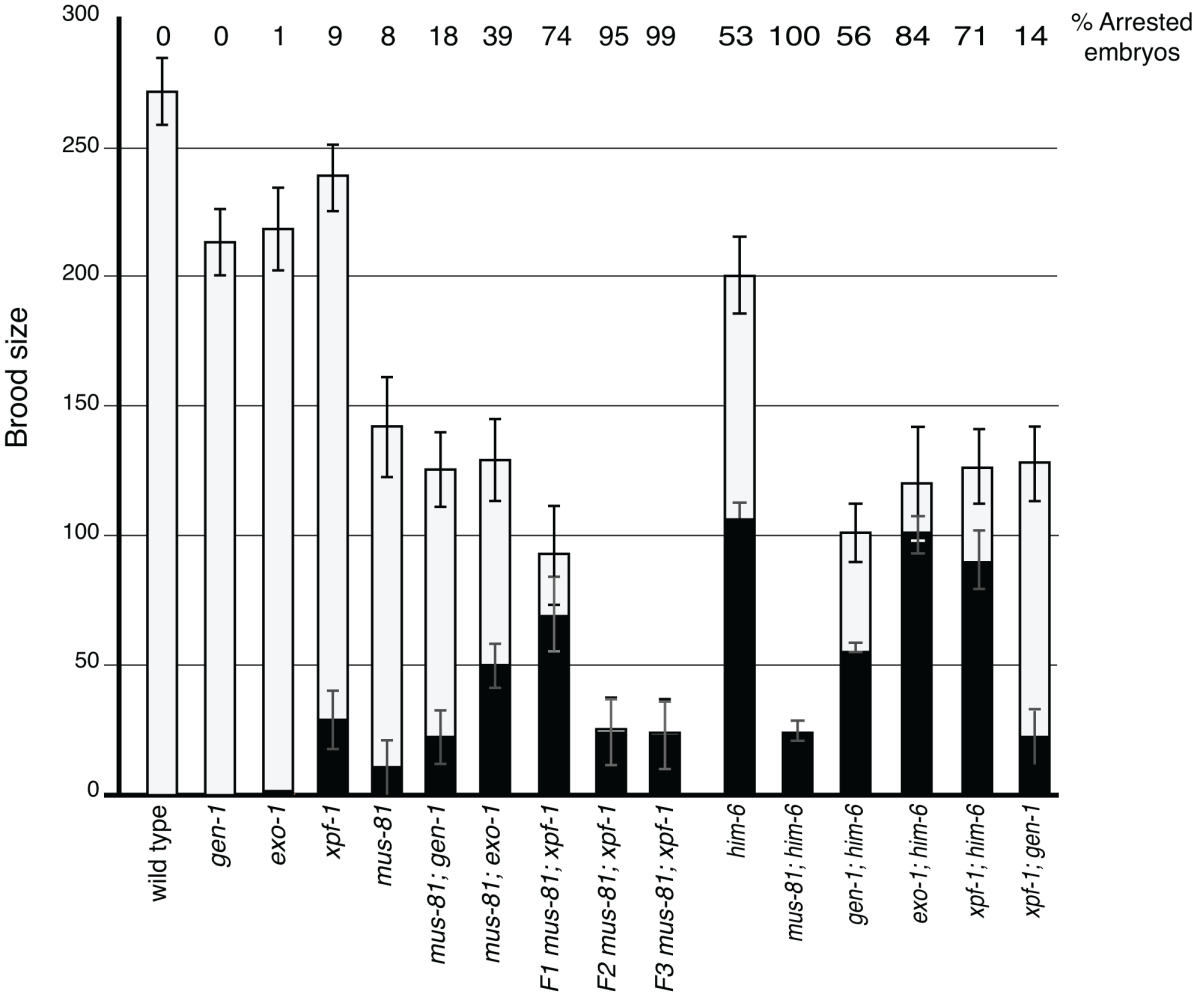
Brood analysis of *mus-81*, *him-6*, *xpf-1*, *exo-1*, *gen-1* and double mutants. White bars – average number of embryos. Black bars – average number of arrested embryos. Error bars are standard error of the mean.

Several recent studies have described redundancy between Mus81 and other related structure specific endonucleases in the resolution of meiotic and mitotic JMs in yeast [14], [36]–[38]. To test whether MUS-81 acted redundantly with other structure-specific nucleases in *C. elegans*, we constructed double mutants with *mus-81* and the *C. elegans* orthologs of the endonucleases, *gen-1* and *xpf-1*, and the endo/exonuclease *exo-1*. Animals lacking GEN-1 or EXO-1 did not show statistically significant differences in brood size, arrested embryos, or frequency of males compared to wild type (Figure 3, Table 1). In contrast to the *gen-1* and *exo-1* single mutants, *xpf-1* mutants produced 9% arrested embryos and 2% males (Student's *t*-test p<0.005), most likely as a result of general chromosome non-disjunction [39]. Loss of *gen-1* or *exo-1* enhanced the phenotype of the *mus-81* mutant, increasing the frequency of arrested embryos from ∼8% in *mus-81* to 18% in *mus-81; gen-1* and 39% in *mus-81; exo-1* (Table 1). In contrast, loss of *xpf-1* in the *mus-81* mutant resulted in a dramatic increase in the frequency of inviable embryos to levels far greater than would be expected for the additive effects of the two mutations (74% vs 16% expected for additive effects of the *mus-81* and *xpf-1*). Furthermore, the *mus-81; xpf-1* double mutant could not be maintained as a homozygous strain. The effect on viability was increasingly more pronounced in the *mus-81; xpf-1* F2 and F3 generations with the brood size decreasing significantly and ∼95% (F2) and ∼99% (F3) of the embryos arresting (Figure 3). In addition, the frequency of sterile animals or animals producing 100% inviable embryos increased from 2 of 23 lines in the F1 generation to 5 of 10 lines in the F2 and 9 of 10 lines in the F3. These data suggested that MUS-81 and XPF-1 have redundant roles in maintaining a functional germ line whereas GEN-1 and EXO-1 did not, either alone or in combination with loss of MUS-81.

The inviability of *mus-81; xpf-1* strains precluded measuring CO frequencies with visible markers in the double mutant so we opted to measure COs in animals homozygous for *xpf-1* and heterozygous for *mus-81*. Strikingly, *mus-81/+; xpf-1* animals exhibited a significant decrease in COs compared to *mus-81/+* animals suggesting that MUS-81 and XPF-1 function redundantly to promote COs (Figure 2). This result is consistent with the decrease in COs observed in *mus-81; xpf-1* double mutants by Saito *et al.* [34] and Agostinho *et al.* [33]. Although the number of COs was reduced in *mus-81/+; xpf-1* animals, *mus-81; xpf-1* mutants did not show a significant difference in the number of ZHP-3 foci in meiotic nuclei (Figure 2B; Figure S3) suggesting that MUS-81 and XPF-1 act downstream of ZHP-3 in meiotic progression.

### Loss of *mus-81* and *xpf-1* does not affect the formation or persistence of RAD-51 foci

**Figure 4:**
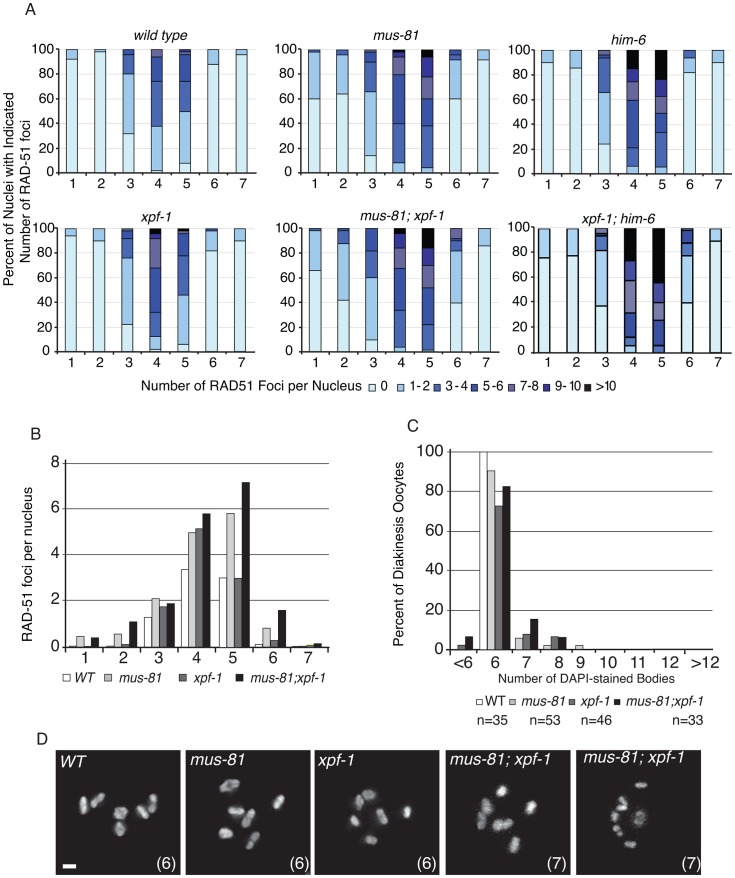
MUS-81 and XPF-1 do not act redundantly to limit the formation of recombination intermediates. (A) Distribution of RAD-51 foci in WT, *mus-81*, *xpf-1*, and *mus-81; xpf-1* animals. Data for WT, *mus-81*, and *him-6* repeated from Figure 1 for ease of comparison. Zone definitions: 1 Early mitotic 2 Late mitotic 3 Transition 4 Early pachytene 5 Mid pachytene 6 Late pachytene 7 Diplotene. (B) Average number of RAD-51 foci per nucleus from A. (C) Average number of DAPI-stained bodies in diakinesis oocytes of WT, *mus-81*, *xpf-1*, and *mus-81; xpf-1* animals. (D) Representative images of DAPI-stained bodies in diakinesis oocytes of WT, *mus-81; xpf-1*, and *mus-81; xpf-1* animals. Bars 2 µm.

To further investigate the phenotypic consequence of losing both MUS-81 and XPF-1, we stained double mutant germ lines with an anti-RAD-51 antibody to observe the progression of early meiotic RIs. We observed that the appearance and subsequent disappearance of RIs was not as profoundly affected in *mus-81; xpf-1* mutant animals compared to *mus-81; him-6* double mutants (Figure 4A,B). RAD-51 foci were only slightly but significantly increased in the mid and late pachytene zones of the *mus-81; xpf-1* double mutant compared to either single mutant (Student's *t*-test p<0.05). Consistent with these observations, there was no measurable increase in DAPI-stained bodies in the *mus-81; xpf-1* double mutant beyond what would be expected from additive effects of the two mutants (Figure 4C,D). Although *mus-81; him-6* and *mus-81; xpf-1* double mutants produced severely reduced broods and increased embryonic arrest, the mechanisms underlying their respective phenotypes appear to be distinct.

### MUS-81 and XPF-1 act redundantly to promote meiotic maturation of bivalents

The lack of obvious early RI defects to account for the inviability of the *mus-81; xpf-1* animals and the decrease in COs observed in *mus-81/+; xpf-1* mutants raised the possibility that MUS-81 and XPF-1 act redundantly to resolve late JMs to produce meiotic COs. To test this hypothesis, we first examined whether loss of MUS-81 and XPF-1 affected the kinetics of CO resolution by monitoring the assembly/disassembly of the synaptonemal complex (SC) component SYP-1. Previous studies in *C. elegans* have shown that either COs or CO precursors trigger the asymmetric dissolution of the SC [40]. SYP-1 is first disassembled in the region between the CO (or CO precursor) and the most distant telomere of bivalent chromosomes in diplotene nuclei. This creates an asymmetry with a long arm of the bivalent that lacks SYP-1 and a short arm that contains SYP-1. Later in diakinesis, the remaining SYP-1 dissociates from the short arms and the Aurora-like kinase AIR-2 becomes concentrated on the short arms. Thus, the maturation of COs during diakinesis can be followed by observing the asymmetric diassembly of the SC and the appearance of AIR-2 on diakinesis oocytes.

**Figure 5:**
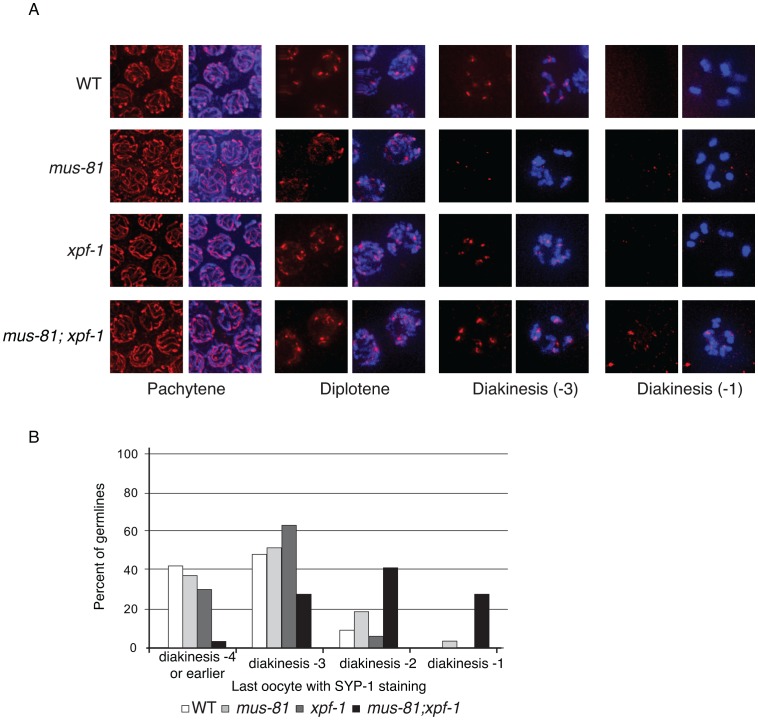
Loss of MUS-81 and XPF-1 affects the maturation of meiotic chromosomes. (A) Representative images of germ-line nuclei stained with an antibody recognizing the core SC component SYP-1. (B) Quantification of last oocyte with SYP-1 staining present.

**Figure 6:**
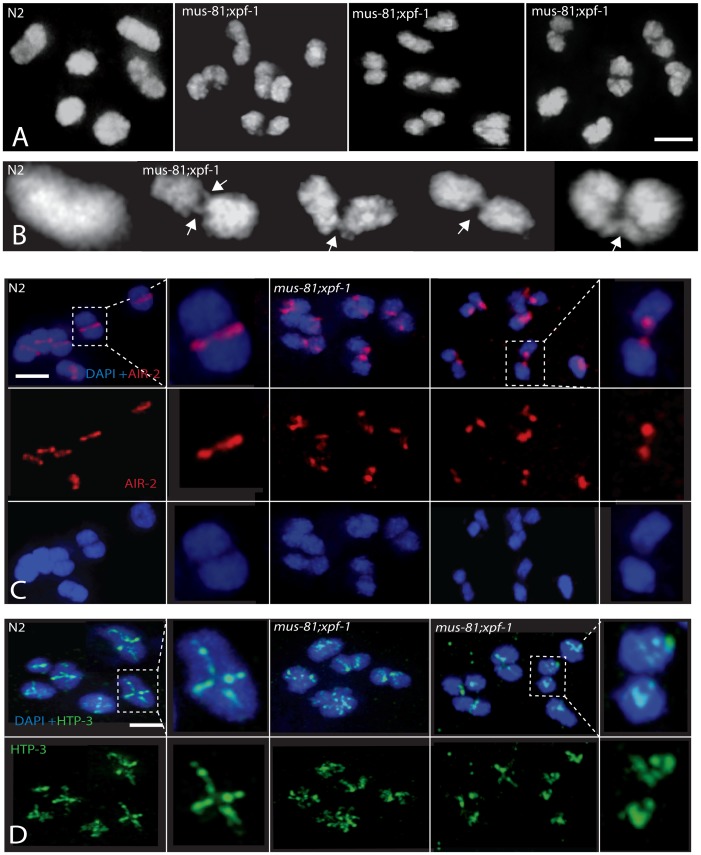
Comparison of diakinesis chromosome bivalents in wild type and *mus-81; xpf-1* animals. (A) Representative DAPI-stained -1 oocytes illustrating differences in the morphology of wild type and *mus-81; xpf-1* bivalents. (B) Representative images illustrating fine DNA bridges between the DAPI-stained bodies in *mus-81; xpf-1* animals. (C) AIR-2 antibody staining in −1 oocytes in wild type and *mus-81; xpf-1* animals. Note how AIR-2 staining flanks the DNA bridge regions. (D) Representative images of antibody staining of the synaptonemal complex axial element protein HTP-3 in −1 oocytes. Note that the bivalents in *mus-81; xpf-1* do not exhibit the wild type cruciform pattern for HTP-3 staining suggesting that the synaptonemal complex and bivalent architecture is disorganized in double mutant. Bars 2 µm.

SYP-1 staining was normal in pachytene and diplotene of all single and double mutants consistent with proper chromosome synapsis and formation of early RIs (Figure 5A). In contrast, *mus-81* and *mus-81; xpf-1* animals exhibited defects later in meiotic progression with the timely disassembly of SYP-1 in diakinetic oocytes. In wild type animals the SC was disassembled in diakinetic oocytes with no visible SYP-1 remaining in the most proximal oocyte (diakinesis −1). In *mus-81* animals, SYP-1 staining persisted in late diakinesis with 20% of −2 oocytes and ∼5% of −1 oocytes containing visible SYP-1 staining. This phenotype was exacerbated in the *mus-81; xpf-1* double mutant with ∼100% of −2 oocytes and 25% of −1 oocytes containing SYP-1 staining (Figure 5B). In addition to the defects in SYP-1 disassembly, bivalents in *mus-81; xpf-1* double mutants also exhibited a highly unusual morphology with DNA bridges present between the two DAPI-staining bodies in each bivalent (Figure 6B). We observed 12 univalents in *mus-81; xpf-1; spo-11* animals, indicating that the DNA bridges in *mus-81; xpf-1* animals were dependent on meiotic DSBs (Figure S4).

In wild type animals, the axial element protein HTP-3 forms a cruciform pattern between sister chromatids along both the short and long arms of the diakinesis bivalent [41] (Figure 6D). In *mus-81; xpf-1* mutants HTP-3 localization on most bivalents was highly disorganized suggesting that their structure was disrupted (Figure 6D). Further evidence for the disruption of bivalents in *mus-81; xpf-1* mutants came from AIR-2 staining. In wild type animals, AIR-2 was localized between the short arms of the sister chromatids in the bivalent, appearing as a single distinct line, whereas in *mus-81; xpf-1*, AIR-2 appeared in two distinct spots on either side of the DNA bridge spanning the two DAPI bodies (Figure 6C).

### Human GEN1 can rescue the meiotic defects of *mus-81; xpf-1* double mutants

**Figure 7:**
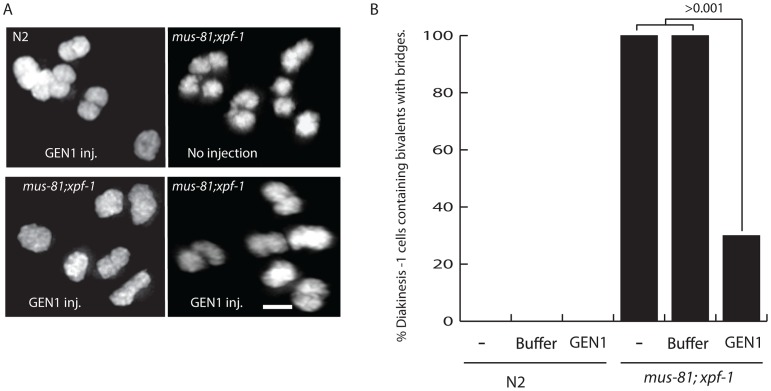
Injection of human GEN1 rescues the fine DNA bridges observed between DAPI-staining bodies of bivalents in *mus-81; xpf-1* diakinetic oocytes. (A) Representative images of DAPI stained wild type and *mus-81; xpf-1* −1 oocytes after germline injection of human GEN1. (B) Quantification of fine DNA bridges in wild type and *mus-81; xpf-1* after injection with either buffer and human GEN1 or buffer only. 20 germlines were scored per condition. Bars 2 µm.

Three lines of evidence support the hypothesis that unresolved late RIs, possibly HJs, were responsible for disrupting bivalent maturation in the *mus-81; xpf-1* double mutant: i) the retarded SYP-1 disassembly in late stage oocytes; ii) the disorganized structure of the axial element and AIR-2 staining in the bivalent; and iii) the presence of a fine DNA bridge between bivalents. To determine if these phenotypes were the result of persistent unresolved JMs, we examined the impact of germline injection of human GEN1 on the presence of DNA bridges between DNA bodies, presumably homologs, in late diakinesis bivalents. Human GEN1 has been previously shown to promote HJ resolution *in vitro* and in *mus81* mutant *S. pombe* strains [8], [42]. Strikingly, germline injection of human GEN1, but not buffer control, significantly reduced the number of nuclei containing bivalents with DNA bridges from 100% to 19% (Figure 7). Taken together, these results suggested that the defects observed in *mus-81; xpf-1* diakinesis oocytes were due to a failure to resolve HJs to produce COs.

## Discussion

The appropriate resolution of meiotic recombination intermediates (RIs) is critical for chromosome segregation at the first meiotic division. Errors during meiotic chromosome segregation can lead to aneuploidy and compromise the faithful transmission of genetic material. Organisms have therefore evolved a number of proteins that can resolve joint molecules (JMs) and in some organisms these resolvases act redundantly to ensure that all JMs are processed before chromosome segregation. This redundancy has made it difficult to identify meiotic Holliday junction resolvases *in vivo*. The complexity associated with Holliday junction resolution is well illustrated by the endonuclease component SLX4 (BTBD12). SLX4 together with SLX1 can resolve Holliday junctions. However, SLX4 also binds to a number of helicases and endonucleases that possess JM processing activity including MUS81, GEN1, BLM, and XPF. This observation has lead to the proposal that SLX4 functions as a platform for the coordination of a number of JM resolving enzymes complicating the analysis of any individual component (for a review, see [43]). It is unknown why JM resolution is so modular, with a great deal of redundancy built into the system. Furthermore, different organisms rely predominantly on different subsets of enzymes to resolve JMs, and it is unclear whether this reflects differences in RIs or whether evolution has shaped the different nuclease preference.

In this study, we set out to define the enzymes responsible for JM resolution during meiosis in *C. elegans*. Unexpectedly, our results implicate that the structure specific endonuclease MUS-81 processes both early and late RIs in *C. elegans*. We propose that MUS-81: 1) functions to limit the formation or persistence of early RIs marked by RAD-51 that form when the *C. elegans* Sgs1 homolog HIM-6 is absent; and 2) acts redundantly with the related XPF-1 endonuclease to resolve late JM intermediates required to produce COs.

### HIM-6/BLM helicase is a key regulator of meiotic recombination intermediates

In budding yeast, Sgs1 regulates the processing of meiotic RIs. Loss of Sgs1 function in meiosis results in an accumulation of JMs that require Mus81 and other enzymes for resolution; when both Sgs1 and Mus81 are lost, unresolved JMs persist into anaphase and cause meiotic catastrophe and death [11], [12]. We found that in the absence of HIM-6, MUS-81 was required to prevent the accumulation and persistence of RIs during pachytene. Based on the large number of RAD-51 foci in *mus-81; him-6* double mutants it appears that most RIs are processed during pachytene by either HIM-6 or MUS-81. It is estimated that there are between 30–65 recombination intermediates formed per nucleus during pachytene [44]–[46]. This assertion is based on RAD-51 foci in *rad-54* mutants, which are compromised for the later steps of homologous recombination downstream of RAD-51 loading onto the processed DSBs. Therefore, 80–90% of all meiotic recombination intermediates are processed to form NCOs by HIM-6, and in the absence of HIM-6, by MUS-81. In the absence of both HIM-6 and MUS-81, early RIs are not resolved and persist resulting in chromosome breakage and inviability. In some cases, fine DNA bridges could be seen between late diakinesis bivalents (data not shown). However, given the large number of unresolved early RIs in *mus-81; him-6* mutants, we could not ascertain whether these bridges resulted from early unresolved RIs that persisted to diakinesis such as multichromatid JMs or if they were interhomolog JMs, as seen in the *mus-81; xpf-1* mutant.

In contrast to MUS-81, XPF-1 does not appear to have a role in processing these early RAD-51-associated RIs. The meiotic phenotype of *xpf-1; him-6* mutant animals was no worse than that expected for an additive effect of the two mutations (Figure 4A). MUS-81 and XPF-1 acted redundantly to resolve late RIs, but not the early RIs that arise in the absence of HIM-6. Moreover, mutation in *mus-81*, but not in *xpf-1*, was synthetic lethal when combined with mutations in the anti-recombinase *rtel-1*. We presume this synthetic lethality reflects a failure to resolve aberrant meiotic RI that form in the *rtel-1* mutant [23]. Consistent with this hypothesis, *mus-81 rtel-1* animals exhibited elevated numbers of RAD-51 foci [47] similar to *mus-81; him-6*. This data suggested that HIM-6 and RTEL-1 act to limit or remove recombination intermediates and in their absence MUS-81 is needed to process these RIs. It is likely that RTEL-1 and HIM-6 have different roles in the processing of early RIs. Loss of RTEL-1 results in an increase in COs, presumably because D-loops are not disassembled leading to an increase in CO-forming RIs, whereas loss of HIM-6 results in a decrease in the frequency of COs [48]. How HIM-6 promotes CO formation in *C. elegans* is not yet clear. In budding yeast, the HIM-6 homolog, Sgs1, is proposed to be the central regulator of JM resolution, directing ∼50% of RIs to NCO outcomes before they form stable JM intermediates and the remaining RIs to CO outcomes, perhaps by preventing intersister and multichromatid JMs thereby ensuring that the remaining RIs result in productive JMs that can be resolved as COs [10], [13]. It is possible that HIM-6 functions similarly in *C. elegans*, since loss of HIM-6 results in an increase in RAD-51-associated RIs in pachytene and as in budding yeast these RIs require MUS-81 for processing. Furthermore, loss of HIM-6 results in a decrease in the frequency of COs.

Similar to MUS-81, HIM-18 (the Slx4 ortholog) is required for wild type levels of crossovers in *C. elegans*. Like *mus-81* mutants, *him-18* mutants exhibit synthetic lethality when combined with mutations in *him-6*, with evidence of increased meiotic recombination intermediates [24]. However, *him-18; him-6* double mutants do not exhibit an increase in DAPI-stained bodies at diakinesis, unlike *mus-81; him-6* mutants. In fact, loss of HIM-18 suppressed the increase in DAPI-stained bodies at diakinesis in *him-6*. This suppression of additional DAPI-stained bodies suggested that the chromosome disjunction phenotype associated with *him-6* mutant animals may be the result of inappropriate processing of RIs by HIM-18-associated endonucleases, leading to premature chromosome disjunction. The multiple binding partners of HIM-18 could account for this difference. Saito *et al.* [34] report a physical interaction between HIM-18 and MUS-81, SLX-1, and XPF-1. Loss of HIM-18 could limit the activity of all three endonucleases resulting in persistent recombination intermediates and a reduction in the number of DAPI-stained bodies. Whereas loss of either MUS-81, SLX-1, or XPF-1 in *him-6* would still allow for endonuclease activity from one of the other endonucleases resulting in inappropriate cleavage and chromosome fragmentation. Overall, our data suggests that HIM-6 and MUS-81 have roles similar to those of Sgs1 and Mus81 budding yeast in the processing of early RIs to prevent the formation of aberrant JMs.

### MUS-81 is required for normal levels of crossing over in *C. elegans*

Mus81 has been implicated in ZMM-independent COs in a number of organisms and as such is required for most COs in fission yeast, which lack ZMM proteins and crossover interference. Mus81 is also required to resolve a subset of COs in budding yeast and for a subset of COs in the murine male germline [5]–[7], [19]. In *C. elegans*, COs are tightly regulated with one crossover occurring on each bivalent [49]. Consistent with strong crossover interference, most COs in wild type animals are thought to be ZMM-dependent [22]. In support of this assertion, six ZHP-3 foci, which mark emerging crossovers, are observed in wild type animals [35]. Previously, we found that ZMM-independent COs could occur in certain circumstances, such as in the *rtel-1* mutant or after the generation of excess COs by ionizing radiation-induced breaks, and that these COs were dependent on MUS-81 [23]. Surprisingly, we have found that MUS-81 and XPF-1 are also required for wild type levels of COs. Our data suggest that there are either significant numbers of ZMM-independent COs in *C. elegans* or that MUS-81, XPF-1, and HIM-18 can resolve ZMM-dependent crossovers.

### MUS-81 and XPF-1 act redundantly to resolve late recombination intermediates

Multiple endonucleases are capable of resolving Holliday junctions, complicating the search for eukaryotic resolvases *in vivo*. It is apparent that in many organisms the resolution of Holliday junctions is buffered by redundant resolvases. For example, in budding yeast there are at least three different endonucleases that can contribute to the resolution of meiotic RIs: Mus81, Yen1, and Slx1/Slx4 [10], [13], [14]. In mice there are at least two pathways for CO resolution: one dependent on Mlh1 and Mlh3 and another dependent on Mus81 [19]. We have found that in *C. elegans*, meiotic JM resolution depends on the redundant activities of MUS-81 and XPF-1. Both single mutants showed relatively minor reductions in COs and in the resolution of early RAD-51-associated meiotic RIs. However, *mus-81;xpf-1* double mutants exhibited severe late meiotic phenotypes in diakinesis oocytes consistent with loss of Holliday junction resolution. Unlike *mus-81; him-6* double mutants, the number of RIs in the *mus-81;xpf-1* mutant germline was not elevated significantly compared to either single mutant, and defects in meiotic progression was not observed until late in meiotic prophase at the onset of diplotene. The asymmetric disassembly of SYP-1, which is triggered by COs or CO precursors, was significantly delayed in *mus-81; xpf-1* double mutants compared to wild type animals (100% vs. 10% of −2 oocytes exhibiting SYP-1 staining, respectively). HTP-3 staining, which marks the axial element, was normal in early meiotic *mus-81; xpf-1* nuclei but was highly disorganized in diakinesis oocytes. These data indicate that bivalent maturation, which occurs in response to CO maturation, is compromised. AIR-2, which is concentrated on the short arm of the bivalent at diakinesis, was also disrupted in the *mus-81; xpf-1* mutant, further supporting the hypothesis that the CO or CO precursor is abnormal. The most striking observation was that the *mus-81; xpf-1* double mutant exhibited fine DNA bridges between the two DAPI-staining bodies of a single bivalent. These bridges occurred between AIR-2 staining regions, supporting the hypothesis that these bridges represent a crossover intermediate; AIR-2 concentration in diakinesis is dictated by CO or CO precursors that act as symmetry breaking events in the *C. elegans* meiotic bivalent [40]. Finally, the most compelling evidence that these DNA bridges are unresolved JMs came from germline injection of the human Holliday junction resolvase GEN1 into *mus-81; xpf-1* double mutants. GEN1 injections rescued the persistent SYP-1 staining on late diakinesis chromosomes (data not shown) and also eliminated the DNA bridges evident in the *mus-81; xpf-1* double mutant. These results are consistent with the ability of human GEN1 to both resolve Holliday junctions *in vitro* [42] and to substitute for Mus81 in promoting crossover formation in fission yeast *mus81* mutants [8]. It is interesting to note that endogenous GEN-1 cannot resolve these meiotic JMs in *C. elegans*. It remains to be determined if this is due to GEN-1 not being active at the appropriate time in meiosis or whether *C. elegans* GEN-1 lacks the ability to resolve these meiotic JMs.

In summary, our data support the hypothesis that MUS-81 and HIM-6 act early in meiosis to limit the formation or accumulation of JMs and that the related structure-specific endonucleases MUS-81 and XPF-1 act redundantly to resolve late JMs, which are likely Holliday junctions, to produce crossovers. Further study into the specific contributions of HIM-6, MUS-81, XPF-1, SLX-1 and HIM-18 will shed light on the nature of joint molecule and recombination intermediate processing, control of crossovers, and evolution of HJ resolution.

## Materials and Methods

### *C. elegans* strains

Strains were cultured as described previously [50]. The strains used in this work include: Wild type Bristol N2, VC193 *him-6(ok412)*, FX1937 *mus-81(tm1937)*, CB1487 *xpf-1(e1487)*, FX2842 *xpf-1(tm2842)*, FX2940 *T12A2.8(tm2940)*, FX1842 *F45G2.3(tm1842)*, DW395 *mus-81(tm1937);T12A2.8(tm2940)*, DW402 *mus-81(tm1937);F45G2.3(tm1842)*, DW116 *mus-81(tm1937);xpf-1(e1487)/mIn II*, DW485 *mus-81(tm1937); him-6(ok412)/nT1[gfp]*, KR4825 *unc-45(e286) dpy-17(e164)*, KR4821 *dpy-17(e164) unc-64(e246)*, *mus-81(tm1937); unc-45(e286) dpy-17(e164)*, *mus81(tm1937); dpy-17(e164) unc-64(e246)*, *xpf-1(e1487); dpy-17(e164) unc-64(e246)*.

### Visible marker meiotic recombination assay

Individual animals heterozygous for visible markers and of the genotypes of interest were plated and transferred daily for four days. In each of the broods, wild type, Dpy, Unc and Dpy Unc phenotypes were scored. Recombination frequencies were calculated as in [48]. Individual male animals heterozygous for visible markers and of the genotypes of interest were mated to tester hermaphrodites homozygous for both visible markers and transferred daily for four days. In each of the broods that contained ∼50% male outcross progeny, wild type, Dpy, Unc, and Dpy Unc phenotypes were scored. Recombination frequencies were calculated as recombinants/total brood.

### Immunofluorescence

RAD-51 immunofluorescence was performed as in [47]. RAD-51 foci in the germlines were assessed when the animals were adults. Foci were counted in 100 RAD-51 positive early to mid-pachytene nuclei. This was done in order to avoid counting earlier nuclei that may not have yet formed meiotic DSBs and later nuclei that may be undergoing apoptosis. Primary antibodies (guinea pig anti-SYP-1, chicken anti-HTP-3, rabbit anti-AIR-2, and guinea pig anti-ZHP-3 (pre-adsorbed against *zhp-3* worms)) were all used under standard conditions (as in [47]) at 1∶250. All secondary antibodies were used at 1∶2500 (anti-rabbit Cy3, anti-guinea pig and anti-chicken FITC). DNA was stained with DAPI (0.5 mg/ml) at 1/500. All images were captured using Deltavision microscopy and images were deconvolved using SoftWorx software (Applied Precision).

### Microinjection

Active C-terminally truncated human GEN1 protein (kindly provided by Steve West), was microinjected into the germline syncytium of adult N2 wild type and *mus-81; xpf-1* double mutant animals at 1 ng/µl. Twenty-four hours after injection, germlines were extracted from surviving worms, fixed, and immunostained as detailed above. 20 germlines were scored for each condition. The Mann Whitney test was used to analyze the different conditions. Gaussian approximation was used for calculation of the indicated P-value.

## Supporting Information

Figure S1 Representative images of whole germ lines from wild type, *mus-81*, *xpf-1*, *him-6*, *mus-81; xpf-1*, and *mus-81; him-6* stained with DAPI (Blue) and anti-RAD-51 antibody (Green).
(TIF)

Figure S2 Representative images of germline nuclei from wild type, *mus-81*, *xpf-1*, *him-6*, *mus-81; xpf-1*, and *mus-81; him-6* stained with DAPI (Blue) and anti-RAD-51 antibody (Green).
(EPS)

Figure S3 Representative images of germline nuclei stained with anti-SYP-1, anti-HTP-3, and anti-ZHP-3 antibodies. (A,B) SYP-1 and HTP-3 staining is grossly normal in late pachytene germline nuclei. (C) ZHP-3 foci formation is not grossly affected in *mus-81; xpf-1* double mutants.
(EPS)

Figure S4 Fine DNA bridges in *mus-81; xpf-1* is dependent on SPO-11 activity. (A) Distribution of RAD-51 foci in *spo-11* and *mus-81; xpf-1; spo-11* animals. Zone definitions: 1 Early mitotic 2 Late mitotic 3 Transition 4 Early pachytene 5 Mid pachytene 6 Late pachytene 7 Diplotene. (B) Average number of DAPI-stained bodies in diakinesis oocytes of wild type, *mus-81*, *xpf-1*, and *mus-81; xpf-1; spo-11* animals.
(EPS)
