# Supplementary figures and images for: Joint Molecule Resolution Requires the Redundant Activities of MUS-81 and XPF-1 during Caenorhabditis elegans Meiosis
Source: PLoS Genet. 2013 Jul 18;9(7):e1003582. doi: 10.1371/journal.pgen.1003582 (PMC3715453; doi:10.1371/journal.pgen.1003582)

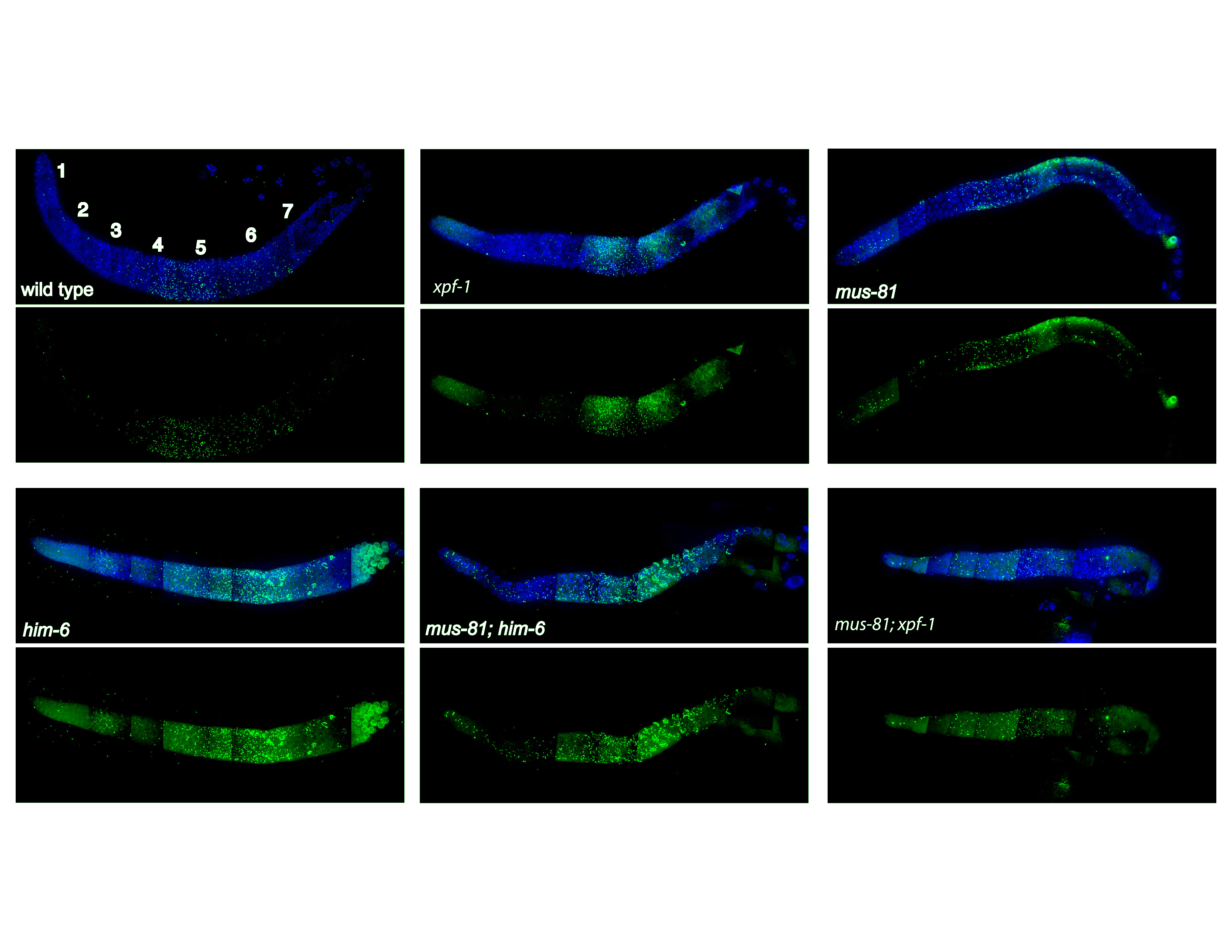

Supplement: Figure S1 — Representative images of whole germ lines from wild type, mus-81, xpf-1, him-6, mus-81; xpf-1, and mus-81; him-6 stained with DAPI (Blue) and anti-RAD-51 antibody (Green). (TIF) [file pgen.1003582.s001.tif]
